# Next generation per- and poly-fluoroalkyl substances: Status and trends, aquatic toxicity, and risk assessment

**DOI:** 10.1016/j.eehl.2022.05.002

**Published:** 2022-07-19

**Authors:** Hannah Mahoney, Yuwei Xie, Markus Brinkmann, John P. Giesy

**Affiliations:** a Toxicology Center, University of Saskatchewan, Saskatoon, Saskatchewan, S7N 5B3, Canada; b School of Environment and Sustainability, University of Saskatchewan, Saskatoon, Saskatchewan, S7N 5C8, Canada; c Global Institute for Water Security, University of Saskatchewan, Saskatoon, Saskatchewan, S7N 3H5, Canada; d Centre for Hydrology, University of Saskatchewan, Saskatoon, Saskatchewan, S7N 1K2, Canada; e Department of Veterinary Biomedical Sciences, University of Saskatchewan, Saskatoon, Saskatchewan, Canada; f Department of Integrative Biology and Center for Integrative Toxicology, Michigan State University, East Lansing, MI, USA; g Department of Environmental Science, Baylor University, Waco, TX, USA

**Keywords:** PFAS, Replacement PFAS, Emerging contaminants, Aquatic toxicity, Chemical scoring

## Abstract

Widespread application of poly- and per-fluoroalkyl substances (PFAS) has resulted in some substances being ubiquitous in environmental matrices. That and their resistance to degradation have allowed them to accumulate in wildlife and humans with potential for toxic effects. While specific substances of concern have been phased-out or banned, other PFAS that are emerging as alternative substances are still produced and are being released into the environment. This review focuses on describing three emerging, replacement PFAS: perfluoroethylcyclohexane sulphonate (PFECHS), 6:2 chlorinated polyfluoroalkyl ether sulfonate (6:2 Cl-PFAES), and hexafluoropropylene oxide dimer acid (HFPO-DA). By summarizing their physicochemical properties, environmental fate and transport, and toxic potencies in comparison to other PFAS compounds, this review offers insight into the viabilities of these chemicals as replacement substances. Using the chemical scoring and ranking assessment model, the relative hazards, uncertainties, and data gaps for each chemical were quantified and related to perfluorooctane sulfonic acid (PFOS) and perfluorooctanoic acid (PFOA) based on their chemical and uncertainty scores. The substances were ranked PFOS > 6:2 Cl-PFAES > PFOA > HFPO-DA > PFECHS according to their potential toxicity and PFECHS > HFPO-DA > 6:2 Cl-PFAES > PFOS > PFOA according to their need for future research. Since future uses of PFAS remain uncertain in the face of governmental regulations and production bans, replacement PFAS will continue to emerge on the world market and in the environment, raising concerns about their general lack of information on mechanisms and toxic potencies.

## Highlights

• Regulations and production bans on legacy PFAS continue to expand.
• Emerging replacement PFAS are rising health, environmental, and regulatory concerns.
• Replacement substances can undergo or show long-range transport potential.
• Novel PFAS bind to nuclear receptors, disrupt metabolism and stress pathways.
• Gaps exist in the (eco)toxic potency and interactions of replacement PFAS.

## Graphical abstract

**Figure:**
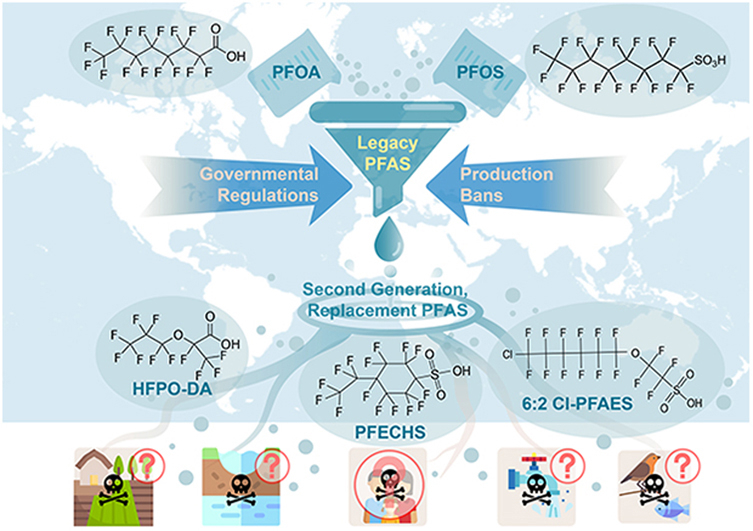

## 1 Introduction

Per and poly-fluoroalkyl substances (PFAS) are a group of industrial chemicals that contain a hydrophobic alkyl chain and a hydrophilic functional group such as carboxylate, sulfonate, or phosphonate [1]. Alkyl chains, which can be straight or branched, consist of one or more carbon atoms in which all or most of the available valence electrons are bound to fluorine (F) atoms [1]. Therefore, PFAS are defined as chemicals with at least one perfluorocarbon moiety (C_n_F_2n_), although structurally, they can differ by the addition of more per-fluorinated (fully fluorinated) or poly-fluorinated chains (partially fluorinated) [1,2].

The presence of multiple strong carbon–carbon and carbon–fluorine bonds not only gives PFAS unique properties and versatility but also means PFAS are stable and resistant to most forms of degradation, including hydrolysis, photolysis, biodegradation, and metabolism [[3], [4], [5]]. This has made PFAS important synthetic chemicals that have been used in a variety of industrial processes and products since the 1950s [[3], [4], [5]]. The hydrophobic and hydrophilic properties of PFAS make them adaptable surface-active substances that repel grease and dirt, adding stain-resistant and hydrophobic properties to fabrics [6]. PFAS have also been used in fire-fighting foams, cleaning supplies, cosmetics, and to reduce the buildup of static electricity in manufacturing electronics, especially microchips [7]. Widespread industrial and commercial applications of PFAS have resulted in some PFAS being ubiquitous in the environment [3,8]. PFAS tend to bind to proteins, resulting in accumulation in plants, wildlife, and humans [1,[8], [9], [10]].

Since the early 2000s, bioaccumulation of PFAS has raised concerns about their potential effects on humans and wildlife. Potential toxic effects of PFAS were discovered in the early 2000s by *Giesy* and *Kannan* after they described for the first time the global extent of PFAS accumulation in marine organisms, terrestrial mammals, and seabirds [3,7,8]. Since then, most research on the effects of PFAS in the environment has focused on two chemical classes of PFAS: perfluoroalkane sulfonic acid and perfluorocarboxylic acids, as well as their anthropogenic precursors [1,7,11]. However, out of these classes and among more than 4700 PFAS, only perfluorooctanoic acid (PFOA), perfluorooctane sulfonic acid (PFOS), perfluorohexanesulfonic acid (PFHxS), and perfluorononanoic acid (PFNA) have been studied extensively [1,7,11].

Of particular concern are the effects PFAS might cause in aquatic environments since lakes, seas, and oceans are often considered environmental sinks of PFAS chemicals [[12], [13], [14], [15]]. After use, PFAS are released into aquatic environments through surface runoff, wastewater effluent, and leaching from products and degradation of precursors [1,15,16]. Environmental monitoring of PFAS in aquatic environments, plants and animals, as well as studies focusing on their effects of exposure, have indicated potential and known toxic effects and potencies of PFAS include reproductive toxicity, growth, and developmental defects, neuro-behavioral defects, and other general disorders arising from the disruption of the immune system and changes in properties of membranes [11].

These known and potential concerns surrounding adverse effects on humans and wildlife have resulted in and continue to result in certain manufacturers voluntarily phasing out production of the legacy substances PFOA and PFOS [[17], [18], [19], [20]]. While PFOA, its salts, and all related compounds were not listed under Annex A of the Stockholm Convention for Virtual Elimination until 2019, its toxicological effects and spread in the environment were known by the public as early as 2004 [3,8]. Conversely, PFOS was listed under Annex B for restriction in 2009 [17]. There has also been a general push in the consumer and stakeholder sectors to virtually eliminate all PFAS ‘forever chemicals’ [18]. Countries globally have begun to implement phase-out plans for legacy PFAS and some second-generation compounds. PFOS and PFOA are regulated along with PFHxS as substances of concern under the European Union (EU) Registration, Evaluation, Authorization, and Restriction of Chemicals (REACH) program [19]. Member states of the EU have often published environmental guidelines for exposure to PFAS that are stricter than those recommended by the EU Environmental Quality Standards, as well as outright banned their use in food packaging paper and cardboard [19]. In Canada, PFOA, PFOS, other long-chain perfluorocarboxylic acids and their salts, and precursors are prohibited, and their addition to the Government of Canada Toxic Substances List has demonstrated the country’s efforts to virtually eliminate their production [20]. While the United States of America (USA) has not yet implemented bans on specific compounds, the United States Environmental Protection Agency (USEPA) has released a PFAS response roadmap and plans leading to the registration of PFOA and PFOS on the Harmful Substances List, and safety guidelines for PFAS exposure are similar to those employed in Canada and the EU [18]. The status of PFAS in the USA largely demonstrates the status of PFAS regulations globally, where outright bans are being discussed or implemented and environmental safety advisories are reported or observed.

However, thousands of PFAS compounds still exist, and compounds with known modes of toxic action are still being manufactured around the globe and available commercially [2]. Due to the complexity, versatility, and number of PFAS chemicals, PFAS will continue to be produced for use in industries that require their unique characteristics and might appear as unintended by-products of industrial processes [[21], [22], [23]]. Recently, attention has shifted to the manufacture of alternatives to replace PFAS, such as PFOS and PFOA, which have been banned or regulated. Although marketed as safer from environmental and human health perspectives, little information exists surrounding the toxicity and environmental fate of these compounds that is available to the general public, and information that is available has yet to be collated in a way that allows robust comparisons of these replacements to legacy substances.

To date, multiple reviews on PFAS have been published covering a range of topics and focuses, including several reviews on the toxicities of legacy PFAS to mammals and humans [[24], [25], [26]], adverse effects of PFAS on aquatic organisms [11,27], and next-steps in the management of PFAS, classifications, and identification [22,28,29]. However, an overview of current knowledge surrounding key next-generation, alternate PFAS in the aquatic environments and their comparative risk assessments were lacking. This review summarizes information on the aquatic toxicity and human risk factors of three emerging replacement PFAS and highlights gaps in information needed for more comprehensive and accurate risk assessments.

Three novel replacement PFAS were chosen as a focus of this review: hexafluoropropylene oxide dimer acid (HFPO-DA, sometimes known as GenX), 6:2 chlorinated polyfluorinated ether sulphonate (6:2 Cl-PFAES, sometimes known as F-53B), and perfluoroethylcyclohexane sulphonate (PFECHS). These three substances were chosen as they represent a broad range of PFAS sub-classes: sulphonates, carbonates, short-chain, and cyclic PFAS [11]. Also, while multiple replacements have been proposed or outlined in research, PFECHS, HFPO-DA, and 6:2 Cl-PFAES have been identified as potential global contaminants with enough toxicity information to relate them to legacy substances [[30], [31], [32]]. Currently known and predicted physicochemical characteristics of these compounds are listed in Table 1.

**Table 1:** Known and predicted physiochemical characteristics of known and emerging replacement perfluoroalkyl substances compared to legacy substances perfluorooctane sulphonic acid (PFOS) and perfluorooctanoic acid (PFOA).

Compound	HFPO-DA	6:2 Cl-PFAES	PFECHS	PFOS	PFOA
Cas #	13252-13-6	756426-58-1	646-83-3	1763-23-1	335-67-1
Structure					
Molecular mass (g/mol)	330.04	300.10	461.13	500.13	414.07
Boiling point (˚C)	129	211	221	249	189
Melting point (˚C)	<40	N/A	74.1	71	55
Partitioning coefficient (Log *K*)	2.84	1.82^a^–3.81	3.19–5.92^a^	4.9	4.81^a^–6.3
Vapor pressure (mmHg)	2.7	0.0268	9.38e-5 to 0.0159^a^	0.0149	0.53
Water solubility (mol/L)	>2.61	1.15e-3	9.68e-6 to 1.35e-3^a^	1.07e-3	7.97e-3
References	(PubChem 114481); [33]	(PubChem 22568738)	(PubChem 101650)	(PubChem 74483)	(PubChem 9554)

a Predicted.

## 2 Methods

**Fig. 1:**
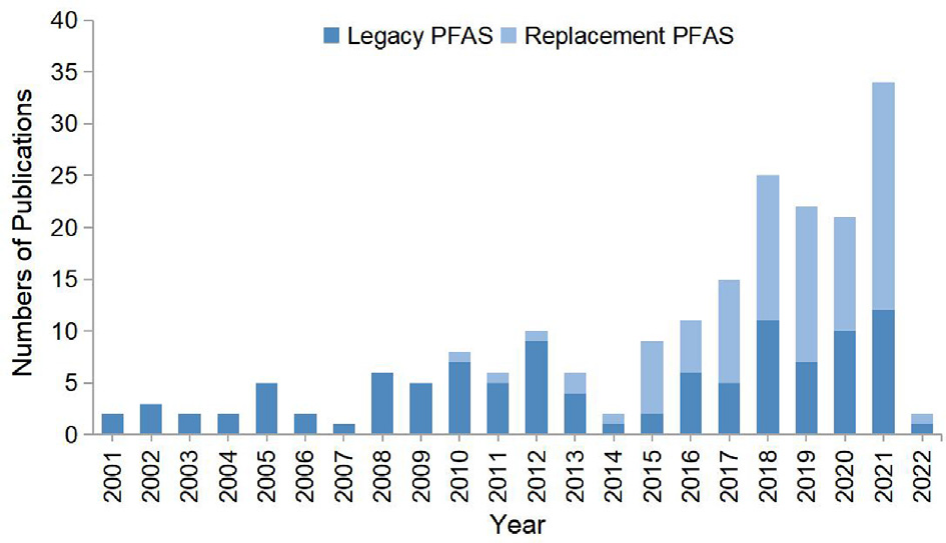
Distribution of the numbers of references cited in this paper is organized by year. This figure also highlights the trend of perfluoroalkyl substance research from mainly legacy perfluoroalkyl substances as indicated by the dark blue bars and numbers of publications to novel perfluoroalkyl substance replacements as indicated by the light blue bars and numbers of publications over time.

Searches of literatures were conducted on Web of Science, Google Scholar, ECOTOX, and PubMed databases using keywords consisting of each chemical name of focus PFECHS, HFPO-DA, and 6:2 Cl-PFAES, the names of highly cited PFAS chemicals (PFOA, PFOS, PFNA, perfluorodecanoic acid (PFDA), perfluorododecanoic acid (PFDoA, PFDoDA)), perfluorodecane sulfonic acid (PFDS), perfluorodecyl phosphonic acid (PFDPA), perfluorohexane sulfonic acid (PFHS), perfluorobutane sulfonic acid (PFBS), perfluoropentanoic acid (PFPA), perfluorotetradecanoic acid (PFTDA), perfluorobutanoic acid (PFBA), perfluoroundecanoic acid (PFUnA or PFUnDA), perfluorooctane sulfamide (PFOSA), perfluorotridecanoic acid (PFTrDA or PFTriA), perfluoroheptanoic acid (PFHpA), perfluoroheptane sulfonoic acid (PFHpS), perfluorohexanoic acid (PFHxA), PFHxS, or perfluorooctylphosphonic acid (PFOPA), toxicity description, regulation status of the chemical, and concentrations in the environment. Identified papers were checked for relevance to aquatic environments, downstream human effects, and environmental concentrations and transport. A total of 188 publications related to legacy and replacement PFAS were selected for inclusion (Fig. 1). Previously published reviews have already synthesized information on adverse effects on fish and aquatic organisms [11]. Therefore, only environmental concentrations, physicochemical properties, human exposure, and adverse outcomes related to the exposure of emerging replacement PFAS of concern, PFECHS, 6:2 Cl-PFAES, and HFPO-DA in the aquatic environment are summarized comparatively.

## 3 Long-distance transport potential and environmental concentrations of emerging replacement PFAS

Primary emission sources of legacy PFAS into the water and air have been identified as industrial facilities producing fluoro-chemicals and wastewater management and treatment facilities [1]. However, even contamination of PFAS in terrestrial environments would be eventually distributed to aquatic environments by abiotic and biotic transfer mechanisms, including advection, dissolution, and biotic uptake [1,24]. Considered a sink for contamination, PFAS partition to the surface water and sediments in aquatic environments [[12], [13], [14], [15]]. While legacy PFAS tend to adsorb to sediments, different substances can be highly mobile, and the log carbon/water partitioning coefficient (log *K*_oc_) of PFAS can range between 0.5 and 5, depending on the substance [34]. In general, shorter chain PFAS remain more soluble in water, while longer chain PFAS adsorb and partition more to sediments. However, the direct measurements of environmental and biological partitioning coefficients of PFAS have proven difficult given their amphiphilic nature and observed behavioral differences compared to other non-ionic polar chemicals [34]. Apart from direct release through industry and waste treatment, PFAS are also known to enter the environment through consumer goods, waste collection sites, and other industrial and consumer processes [[35], [36], [37]].

Multiple studies have indicated that HFPO-DA, 6:2 Cl-PFAES, and PFECHS follow similar pathways of exposure in environments as legacy PFAS [38,39]. The ammonium salt HFPO-DA is a short-chain, organo-fluoride chemical developed to replace PFOA [[40], [41], [42], [43]]. HFPO-DA is often referred to as GenX. For the purpose of this review, GenX will refer to the group of chemicals used in the production of HFPO-DA, such as 2,3,3,3-tetrafluoro-2-(heptafluoropropoxy) propanoic acid and ammonium 2,3,3,3-tetrafluoro-2-(heptafluoropropoxy) propanoate, and will only be used when studies investigating general GenX chemicals are discussed [44,45]. A suspect screening and inter-year comparison of surface waters and sediments within and surrounding the Xiaoguang River, which received wastewaters from a fluoro-chemical production plant in China, identified HFPO-DA, as well as numerous chemicals that were also potentially under the GenX classification [46]. While the concentrations of GenX chemicals were determined to be 1 to 2 orders of magnitude less than those of PFOA, the GenX chemicals followed the same pathways of transport, including horizontal transport in the water, showed no evidence of degradation, and illustrated a tendency to adsorb to sediment [46,47]. It was concluded that GenX chemicals identified in this study posed a similar potential for exposure to humans [46,47]. These findings have also been supported by similar studies, which have quantified downstream concentrations of HFPO-DA and PFOA in waters near fluoro-chemical processing plants throughout Asia and in Europe [[47], [48], [49]].

Known by the trade name F-53B, 6:2 Cl-PLAES is an ether-sulphonate used widely as an alternative to PFOS as a mist-suppressant in the electroplating industry [50,51]. The motivation for its creation is largely attributed to increasing regulations of PFOS, and in China specifically, the lack of regulations on 6:2 Cl-PFAES led to an estimated annual usage of 30–40 t of alternative mist-suppressants in 2009, eventually leading to the detection of 6:2 Cl-PFAES in the aquatic environment [51,52]. The annual release of 6:2 Cl-PFAES is similar to that of PFOS and PFOA, which had an approximate annual release of 62 and 36 t in 2017, respectively [53]. Research on the environmental distribution and transport of 6:2 Cl-PFAES has also indicated that it follows similar pathways of transportation, emission, and degradation as PFOS [30]. 6:2 Cl-PFAES has been found globally in multiple environmental matrices, including the atmosphere, fresh and salt surface waters, cultivated and uncultivated soil, sediment, and drinking water at similar concentrations to PFOS. For example, 6:2 Cl-PFAES is found in concentrations up to 30 ng/L in local Chinese freshwater and PFOS typically around 15 ng/L [30,54].

However, unlike PFOS, only a small percentage of annual emissions of 6:2 Cl-PFAES (0.2%–0.5%) reaches the Arctic by oceanic advection [30]. While it is believed that the bulk of 6:2 Cl-PFAES remains in northern temperate regions not far from its sources in the Eastern hemisphere, a limited number of samples from Europe and North America have contained quantifiable concentrations of 6:2 Cl-PFAES, from 0.01 ng/L to 0.08 ng/L, and up to 52 ng/L near local manufacturing plants [49]. Average concentrations in Chinese freshwater samples ranged from 2 ng/L to 29 ng/L, but local concentrations of 6:2 Cl-PFAES in Chinese freshwater near chromium-plating plants were predicted to reach 2.3 mg/L by 2020, increasing from 0.7 mg/L in 2015 [30]; however, this prediction was not confirmed by the time this review was written. While annual global emissions of 6:2 Cl-PFAES have remained stable (around 12 t), it is predicted to increase as PFOS continues to be phased out and more regulations are introduced [30].

PFECHS is an 8-carbon cyclic PFAS marketed for use as an erosion inhibitor in aircraft hydraulic fluids [55,56]. While the production of PFECHS was voluntarily phased out in the United States *via* 3M’s phase-out of PFOS-based materials beginning in 2002, PFECHS is still permitted to be used in hydraulic fluids by Canada and the United States [55,56]. Besides, PFECHS is neither considered by the Stockholm Convention of Persistent Organic Pollutants to be a PFOS-related substance nor is it proposed as a chemical for listing under the convention [17]. Therefore, PFECHS has continued to be used in various commercial products from manufacturers other than 3M [57]. While the total release of PFECHS into the environment remains largely unreported, Italy reported low release in 2005 at less than 1 t [58]. However, PFECHS has been found in surface waters from the Great Lakes and other freshwater bodies (0.16–5.7 ng/L), predator fish from the Great Lakes (up to 3.7 ng/g wet body weight), the Baltic Sea, samples of drinking water, and within multiple media from the high Arctic [55,[59], [60], [61], [62], [63]]. Detectable concentrations of PFECHS have also been measured in herring gull eggs from the Great Lakes and in liver samples from marine mammals such as ringed seals [64]. Within pooled serum samples from Swedish women, PFECHS has been detected, and concentrations followed throughout generations, suggesting an inter-species bioaccumulation potential of PFECHS exists and could become a potential human health concern [[64], [65], [66]].

The detection and spread of PFECHS are similar to that of PFOS, which has been detected in marine, freshwater, and terrestrial environments, as well as avian, aquatic, and terrestrial organisms [3,8]. While wastewater treatment plants have been associated with the detection of PFECHS in both nearby fish [67] and effluent [68], the greatest and most reliable concentrations have been detected near airports [69,70]. For example, PFECHS detected in runoff water from the Beijing International Airport was measured up to 195 ng/L, but the total amount of PFECHS, its isomers and related impurities can reach up to 324 ng/L [70] (Table 2). Depending on the source measured, the concentrations of PFECHS can be higher than those of PFOS measured from the same sample [64]. PFECHS remains a substance of concern, given it shares many physicochemical properties with PFOS. The compounds have similar molecular masses, boiling points, melting points, and partitioning coefficients (Table 1) [55,56,62].

**Table 2:** Concentrations of replacement PFAS in the Environment.

Compound	Matrix	Concentration	Reference
HFPO-DA	Freshwater	0.1–0.8 ng/L	[49,75]
	Drinking water	1.4–8.0 ng/L^b^	[76]
	Wastewater	Up to 40,000 ng/L^c^	[33]
	Sediment	>100 pg/g	[71]
	Plant material	1–27 ng/g ww^b^	[76]
6:2 Cl-PFAES	Freshwater	<0.01–50 ng/L	[77]
	Drinking water	<0.01–50 ng/L	[77]
	Marine	0.21–7.9 ng/L	[78,79]
	Wastewater	7600 ng/L 65000–120000 ng/L (influent) 43000–78000 ng/L (effluent)	[77,78]
	Sediment	200 pg/g–0.013 ng/g	[71,80]
PFECHS	Freshwater	0.16–5.7 ng/L 20 ng/L^a^	[39,59,60,69]
	Drinking water	4 ng/L	[73]
	Marine	0.043–0.14 ng/L	[62]
	Wastewater	10–195 ng/L	[68,74]
	Sediment	0.0004 ng/g >10 pg/g	[59,61,71]
	Ice cap	<1 ng/L 0.031 ng/mL	[59,61]

a Within 1.61 km of an airport.

b Within 25 km of a fluoropolymer production plant.

c Direct industrial effluent.

To fully answer whether these replacement compounds can be considered global pollutants, potential sources of contamination other than direct and local contamination were taken into consideration. While HFPO-DA was determined to follow similar transport as PFOA in water [46,49,54], this transport was dependent on direct release from processing plants into the environment. However, machine models and published literature have associated HFPO-DA with a high risk of atmospheric deposition [31,32]. While no published studies to date have detected HFPO-DA in remote environments such as Polar regions, it is considered to have the potential to spread to such environments by long-range transport processes [31,32]. HFPO-DA has also been detected in the environment in North America, Europe, and China [46,48,71]. As their industrial application determines whether these compounds become global contaminants through use and release, the probability of HFPO-DA being confirmed as a global contaminant will continue to increase as its usage increases.

Furthermore, while only a small percentage of 6:2 Cl-PFAES is carried by oceanic advection to remote locations [30], it has been detected up to 0.27 ng/g in the livers of polar bears, killer whales, and ringed seals from Arctic environments [49,50], similar to that of PFOS. Environmental concentrations of 6:2 Cl-PFAES have also been shown to be correlated with those of PFOS [49]. Even if 6:2 Cl-PFAES appears to only have a limited ability to travel to the Arctic by oceanic advection, other transport processes such as atmospheric deposition should be further investigated [30]. The detection of 6:2 Cl-PFAES in marine mammals from remote locations is a concerning sign of its potential for long-range transport.

The detection of PFECHS in freshwater lakes has been attributed to direct contamination from local airports, where PFECHS-containing fluids are heavily used [59]. However, PFECHS has also been detected in remote marine/arctic environments without an obvious source of contamination nearby [61,62]. Evidence for long-range transport of PFECHS was outlined by *MacInnis* et al., who proposed oceanic transport processes as the source of PFECHS on the Devon Ice Cap [61]. The detection of PFECHS in the Baltic Sea [62] also supported this hypothesis. However, it was also stated that long-range transport of PFECHS could be due to leakage from commercial airplanes into the atmosphere, but this hypothesis was admittedly challenging to corroborate given the complexity of aviation sources [62]. Mechanism aside, the detection of PFECHS in such remote locations provides support for its referral as a potential global contaminant. Further, PFECHS is considered one of the more widespread PFAS detected in the environment [72].

## 4 Human exposome of emerging replacement PFAS

Detection in human tissues is an important aspect of toxicology testing when completing a risk assessment as it confirms whether humans are a receptor of environmental exposure. Since PFAS as a class are considered to have the potential to bioaccumulate in biota included in human food chains [81] and specific substances such as PFOS and PFOA have been detected in human serum samples at concentrations as high as 44.7 and 10 μg/L, respectively [82], it is important to review whether alternative and replacement PFAS substances also pose this risk. This section will review current known information pertaining to the detection of replacement PFAS in human samples.

While HFPO-DA has been detected in environmental matrices and locations where humans were exposed [76], it has not yet been detected in tissues of humans [83,84]. In a study that aimed to identify novel fluoroethers and legacy PFAS in serum samples from residents residing near or who had lived near a fluoro-chemical processing plant, GenX fluoroethers were not detected with a limit of detection (LOD) of 2 μg/L [84]. Failure to detect HFPO-DA as well as other GenX fluoroethers in human tissues is consistent in studies investigating concentrations in serum and urine of participants who had been exposed to GenX compounds in their drinking water [83,85]. However, these studies consistently employed detection limits at the part per billion (μg/L; ppb) range, although PFAS can commonly be detected at the part per trillion (ng/L; ppt) concentrations in the sources of drinking water [83]. Although it is believed HFPO-DA is effectively eliminated from human bodies given its lesser bioaccumulation potential than other legacy PFAS, HFPO-DA has been shown to be potentially toxic to humans by many toxicity tests, including those with rats, mice, and zebrafish [[86], [87], [88], [89], [90], [91]]. Acute and chronic reference doses for human exposure were calculated by the Environmental Protection Agency to be 30 ng/(kg·day) for acute exposure and 3 ng/(kg·day) for chronic exposure [92]. This is similar to the calculated reference doses for PFOA, which correspond to 20 ng/(kg·day) for sub-chronic exposure [93].

No quantifiable concentrations of 6:2 Cl-PFAES have been detected in the blood plasma of humans in Europe or North America (LOD 0.9 pg/mL–0.5 ng/mL) [94,95]. This result was expected since 6:2 Cl-PFAES is not officially used in Europe and given the small potential for long-range transport of 6:2 Cl-PFAES, as illustrated by *Ti* et al. [30]*.* However, that is not to say that 6:2 Cl-PFAES will not be detected in human samples on these continents in the future, given a limited number of environmental detections in river waters in Europe, and detection in marine mammals from remote locations [50]. Alternatively, 6:2 Cl-PFAES has been detected in the blood serum of people from China at concentrations second to that of PFOA and PFOS (LOD 0.02 ng/mL) [77,96].

Concentrations of 6:2 Cl-PFAES in human blood plasma as great as 0.14 ng/mL have been reported and were greatest in people considered obese [96]. Concentrations detected in serum increased with age, suggesting a high bioaccumulation potential and long half-life in humans [96]. Males also had slightly greater concentrations than did females [96], which supports findings from other PFAS such as PFOA and PFOS [97]. The concentrations of 6:2 Cl-PFAES have also been reported as being comparable to those of PFOA, both in maternal blood sera and cord sera in pregnant women from China, as great as 0.6 ng/mL (LOD 0.01 ng/L) [[77], [78], [79]]. In addition, multiple studies investigating human exposure in China to 6:2 Cl-PFAES have indicated that it is bio-accumulative with a potentially longer half-time in humans than PFOS and PFOA. The log *K*_ow_ and predicted bioaccumulation factors (BAF) of 6:2 Cl-PFAES were 5.29 and 3.81, respectively, compared to 4.49 and 3.28 for PFOS [98,99]. In humans occupationally exposed to 6:2 Cl-PFAES, detected concentrations in blood serum have been reported as great as 5000 ng/mL (LOD 0.01 ng/L) [77]. These results suggest that humans are as susceptible to 6:2 Cl-PFAES exposure and accumulation as they are to PFOS and that 6:2 Cl-PFAES shows the same potential to cross the blood–brain and blood–placenta barrier [78,79,98,99].

Suspect screening has identified PFECHS in pooled human blood serum, cord sera, and placental tissue taken from expecting mothers from Europe at concentrations ranging from 21 ng/L to 38 ng/L (LOD 0.25 ng/mL) [66,95,100]. Converse to 6:2 Cl-PFAES, PFECHS has not yet been reported in tissues of humans in China, likely because it has not until recently been a target of concern, but the detection of PFECHS in drinking waters from China and around the globe suggests that it could be identified in targeted analysis of human blood plasma and sera as well as other tissues [10,59,70,101].

## 5 Aquatic toxicology of legacy PFAS

Legacy PFAS are often not considered acutely toxic relative to other aquatic contaminants found in the environment [25], and concern surrounding their environmental effects is related to their bioaccumulative ability and long half-lives [1,7,11]. In aquatic organisms, the bioaccumulation potential of legacy substances depends on the species exposed and can range from a low potential to a very high potential [81]. In regard to PFOA, serum bioconcentration factors (BCFs) ranged from 9.4 to 578 when calculated in carp (*Cyprinus carpio*) and black rockfish (*Sebastes schlegeli*) [102]. However, the whole body log BCF of PFOA measured across species was only determined to be as high as 1.36, which corresponds to a BCF value of 22 [81]. PFOS is considered to have a BCF as high as 26,000 when whole-body concentrations were measured in catfish (*Lctalurus punctatus*) and large-mouth bass (*Micropterus salmoides*) [27]. In a critical review of the calculated bioaccumulation potential of a number of legacy PFAS, whole body log BAF ranged from 1.30 to 4.86 depending on the substance under study [81]. These values correspond to log BAFs ranging from 3.6 to 4.6 [56]. The bioaccumulation potential of legacy PFAS is one of the defining aspects of their chemical class and allows organisms exposed to low concentrations to accumulate a toxic internal dose [27].

Because a comprehensive review on the adverse effects of PFAS in aquatic environments has already been published [11], this review only briefly describes and summarizes the known effects of PFAS on aquatic receptors, particularly in the domains of the non-targeted and targeted tissue and organ-level effects, and population-level effects. Because toxic potencies of emerging replacement PFAS are largely unknown, the following sections will be used as a foundation for comparing the known effects of legacy PFAS and emerging replacements.

### 5.1 Non-organ-directed bioactive effects of PFAS exposure

Exposure of fish and other aquatic organisms to PFAS can result in both non-organ-directed toxicity and target organ toxicity. Non-organ-directed toxicity can be summarized as toxic effects and potencies relating to oxidative stress and the metabolism of xenobiotics and key macromolecules [11,103]. Several previous studies have identified oxidative stress in aquatic organisms following exposure to PFAS. In a study in which cultured hepatocytes of Nile tilapia (*Oreochromis niloticus*) were exposed to 30 mg/L of PFOS and PFOA, increased activities of superoxide dismutase (SOD), catalase (CAT), and glutathione reductase were observed, suggesting greater concentrations of reactive oxygen species (ROS) [104]. Similarly, exposure of zebrafish embryos (*Danio rerio*) to 1 mg/L of PFOS resulted in ROS production and induction of antioxidants [105]. The Results of these and other studies have suggested that the production of antioxidants after exposure to PFAS is related to the activation of the mitogen-activated protein kinase pathway [105,106]. For example, studies investigating the effects of exposure of zebrafish larvae or embryos to PFNA or PFOS have found an increased abundance of transcripts coding for kinases and transcription factors involved in the mitogen-activated protein kinase signaling pathway, such as jun-N-terminal kinases, and nuclear respiratory factors (NRF-1 and NRF-2) [[105], [106], [107], [108], [109]].

Exposure to PFAS also alters the expression and regulation of genes related to the metabolism of xenobiotics. In fish, PFAS have been shown to upregulate the expressions of various phase I cytochrome P450 enzymes as well as phase II detoxification enzymes and phase III transporter receptors [110,111]. The upregulation of cytochrome P450 genes, such as CYP3A and CYP2Y3, was observed in male cryptid fish (*Gobiocypris rarus*) exposed to 30 mg/L of PFOA [112]. Significant induction of CYP3A has also been observed in other fish exposed to PFOA, such as rainbow trout (*Oncorhynchus mykiss*) [113,114]. In addition, exposure to PFAS can result in the activation of the aryl hydrocarbon receptor (AhR), peroxisome proliferated activated receptor (PPAR), and the pregnane X receptor (PXR), which has been demonstrated by an increase in transcription abundance of some genes in a variety of species exposed to PFOS, PFOA, and mixtures containing each [111,115]. Extensively described by Lee et al., these findings suggest that organisms attempt to excrete PFAS by activating the PPAR, PXR, AhR receptors, and by the use of biotransformation mechanisms that involve phase I (cytochrome P450), phase II (glutathione), and phase III (ATP-binding cassette) enzymes [11]. The activation of PPAR, AhR, PXR, and other receptors, including the retinoic acid receptor, recombinant retinoic X receptor, and liver X receptor by PFAS, has also been shown to affect the metabolism of lipids and carbohydrates in aquatic species [111,[116], [117], [118], [119]].

### 5.2 Target-organ and -system bioactive effects of exposure to PFAS

Exposure to PFAS has been associated with endocrine-disrupting effects, including significant regulatory changes in genes connected with serum testosterone, 17β-estradiol (E2), and production of the egg yolk protein, vitellogenin [113,120,122,123]. In the brain, gonads, and liver of zebrafish, significant changes in transcription abundance of genes for the follicle-stimulating hormone receptor, luteinizing hormone receptor, and the steroidogenic acute regulatory protein were observed after exposure to 1 mg/L of PFNA [123]. In fathead minnows (*Pimephales promelas),* exposure to PFOS resulted in greater concentrations of plasma testosterone [124]. These studies have provided evidence that PFAS can directly bind with receptors along with the hypothalamus-pituitary-gonad-liver axis and estrogen receptors (ERs) [11] and are supported by observed tissue and organ level effects in affected organisms. A study investigating the exposure of cryptid fish to 30 mg/L of PFOA reported degenerating oocytes [112]. Similar results have also been reported by later studies that observed ovarian follicle cell atrophy, degeneration, and spermatozoa paucity in fish exposed to PFOA and mixtures of PFOS, PFOA, PFNA, and PFBS [11,125].

The disruption of thyroid function has also been observed in aquatic organisms exposed to PFAS. Exposure to PFDoA has resulted in a number of transcriptional changes, such as the upregulation of genes like thyrotropin-releasing hormone*,* corticotropin-releasing hormone*,* and iodothyronine deiodinase *2,* a gene that codes enzymes important for the activation and de-activation of thyroid hormones in zebrafish [120,126]. The downregulation of genes such as thyroglobulin *(Tg)* and thyroid hormone receptor *(THRβ)* has also been observed concurrently with the above gene upregulation in zebrafish [120]. Similar results were not only observed in zebrafish exposed to PFOS but also included the upregulation of early development-related genes necessary for the differentiation and formation of thyroid follicles such as homeobox protein *(Hhex)* and paired box gene 8 *(PAX8)* [127]. Concurrent observed changes in thyroid structure and function were also observed in accordance with the above molecular-level changes [127,128]. Significant changes such as the inhibition of growth and decreased concentrations of thyroid hormone have been observed in zebrafish exposed to either PFOS or PFDoA [126], and exposure to mixtures including PFOS, PFOA, PFNA, and PFBS have resulted in thyroid follicle cell degeneration and atrophy of male fish [129].

Studies investigating the effects of PFAS have suggested the hat accumulation of lipids in the liver is a primary outcome of PFAS exposure [113,116,118,119]. The previous discussion on molecular and transcriptomic changes in aquatic organisms has suggested that PFAS disrupt lipid metabolism. These findings, along with tissue- and systemic-level analyses, have linked PFAS exposure with lipid metabolism-related hepatoxicity [116]. In zebrafish chronically exposed to 0.5 μM (0.3 mg/L) of PFOS, serum cholesterol content measured as the low- and very-low-density lipoprotein ratio was decreased along with a lesser ATP content in blood serum [118]. In contrast, total cholesterol and glycerol contents were greater in larger livers, which suggested an accumulation of lipids in the liver [118]. Hepatocyte viability was also decreased in Nile tilapia exposed to PFOS or PFOA [130] and in zebrafish exposed to PFOA, PFBA, or PFHxA [130]. The accumulation of lipid droplets in the liver, and swelling of hepatocytes, and hepatocellular vacuolar degeneration have also been observed in fishes, such as zebrafish and cryptid fish exposed to PFOS, PFOA, or PFDoA [116,118,119,121]. Steatosis (fatty liver) was observed in zebrafish exposed to 0.3 mg/L of PFOS [118] and research into the molecular responses matched those observed in mammals [116]. Lipid accumulation was also observed in adult zebrafish after chronic exposure to 0.3 mg/L PFOS and observed brittle and pale livers in PFOS-exposed fish compared to the soft and sanguine livers of control fish suggested liver degeneration [121].

The main mechanism associated with PFAS-induced hepatoxicity is the ability of PFAS to bind to proteins such as serum albumin [99], fatty acid protein [103], and apolipoprotein A-Ⅰ [119,131]. While binding to serum albumin is typically observed in mammals, binding into fatty-acid proteins in fish livers and apolipoproteins have the potential to alter liver metabolism as described above, leading to hepatoxicity and associated apical events [99,103,119]. However, apical events related to protein binding of PFAS were substance-dependent, as only some resulted in moderate biochemical and molecular effects at concentrations higher than those found in the environment. In a study that investigated changes in fathead minnow exposed to PFOA, biochemical endpoints such as altered fatty-acid oxidase were observed at concentrations of 1 and 30 mg/L [99]. In another study that identified alterations in apolipoprotein genes in rare minnows (*G. rarus*), only concentrations around 10 mg/L resulted in an altered expression [119]. Therefore, the severity of the effect PFAS have on the liver is dependent on the substance of exposure, and, in the case of substances like PFOA, can be relatively non-toxic at environmentally relevant concentrations [99].

The effects of PFAS on the metabolism of lipids, as well as the general amphiphilic nature of PFAS, are also associated with altered cellular membranes [[132], [133], [134], [135], [136]]. Exposure of Atlantic cod (*Gadus morhua)* to the mixtures of PFAS caused the enrichment of poly-unsaturated acyl-chains in phospholipids along with the perturbation of lipid metabolism [136]. Acyl-chains confer membrane flexibility, enabling density adjustments that are theorized to be in response to acute membrane deformations potentially caused by PFAS exposure [136]. Previous studies have also demonstrated that exposure to PFOS results in increased membrane permeability and fluidity and decreased membrane potential [133].

Based on the targeted and non-targeted molecular and organ level responses of aquatic organisms, several molecular and cellular biomarkers of toxicity of PFAS have been suggested. These biomarkers include changes in the expressions of apolipoprotein (*ApoAL, ApoALV)* due to its specific role in lipid metabolism, serum lipid content, liver triacylglycerol content, lipid droplet content, and the hepatosomatic index (HSI) due to the ability of PFAS to influence the accumulation of lipids *via* changes in synthesis, uptake, and β-oxidation [11]. Changes in expressions of some key nuclear receptors, such as PPAR, THR, liver X receptor, and PXR, could also be used as biomarkers for PFAS exposure. However, they lack specificity across species and experiments [11]. While not specific to PFAS exposure, genes for xenobiotic metabolism and oxidative stress are still consistently affected, and specific genes such as *CYP3A1,* jun-N-terminal kinases*,* and *Nrf2* are important to characterize the molecular effects of exposure [11,25,137]. At the cellular level, altered amounts of glutathione (GSH), SOD, CAT, and lipid peroxidation (LPO) in the liver can also be used to characterize and mark PFAS exposure effects [11,25,137].

### 5.3 Individual- and population-level responses to PFAS exposure

Molecular and mechanical alterations in response to exposure to PFAS can cause abnormalities in growth and development, as well as altered endpoints in reproduction and behavior [6,11]. These can include reductions in fecundity of the parent generation [124], as well as decreases in hatching rates, larvae survival, body length, and developmental abnormalities [127]. Multiple studies have demonstrated similar results, which observed decreases in larval survival and sperm density in male zebrafish exposed to PFOS [138]*.* The fecundity of Japanese medaka (*O. latipes)* was significantly decreased with exposure to a mixture of PFOS, PFOA, PFNA, and PFBS [129]. The results of such studies have suggested the potential for population-level effects of PFAS, particularly PFOS, which include a greater ratio of female fish as well as decreases in population numbers [138].

However, some studies have reported that certain PFAS do not cause reproductive toxicity in some species of fish. A study investigating zebrafish exposed to PFOA showed no significant changes in hatching rates, fecundity, or fertility [120]. Although reductions in fecundity of the parental generation were observed when exposed to 0.3 mg/L PFOS, there were no significant changes in the hatching rates of eggs or effects on the growth and development of their offspring exposed to up to 0.3 mg/L of PFOS [124]. As well, investigations into aquatic invertebrates often lead to more contrasting results. In a study that investigated the effect of acute and chronic exposure of PFOA and other short-chain substances perfluorobutanoic acid (PFBA), and PFHxA on the mortality and fecundity of *Daphnia magnia*, PFOA was demonstrated to cause marked decreases in reproductive rates and increases in mortalities, where the calculated effective concentration (EC50) of 239 mg/L was significantly lower than that of PFBA and PFHxA which had EC50’s of 5251 mg/L and 1048 mg/L, respectively [139]. Such differences in the toxicity of PFOA on fecundity across species highlight how PFAS research requires a broad range of studies on different endpoints and species to create a robust understanding of their effects on environmental populations.

The growth and development of aquatic organisms could also be affected by PFAS due to underlying mechanisms related to oxidative stress, thyroid disruption, and development-related gene regulation [11,126,127]. In a study by Zhang et al. [126], exposure to 6 mg/L of PFDoA inhibited growth and caused spine deformities in larval zebrafish, likely due to the disruption of thyroid function. Along with the upregulation of genes, such as *PAX8* and *Hhex,* zebrafish embryos exposed to 5 mg/L of PFOS were characterized by significant morphological abnormalities and developmental toxicological effects [127]. Underlying mechanisms affecting development might also be linked to neurobehavioral changes associated with PFAS exposure. In zebrafish exposed to PFDoA, a decrease in swimming speed was observed, along with a reduction of acetylcholine content (ACh) [140]. This suggested that ACh enzyme activity could have been inhibited by PFAS, which then resulted in the reduction of ACh [140]. Reduced behavioral activity has also been observed in goldfish exposed to PFOS [141]. This observation is supported by a reduction of aggressive behavior in male zebrafish exposed to PFOS and other PFAS [142]. However, some studies have also reported conflicting behavioral results. In zebrafish exposed to PFOS, there was a significant increase in basal swimming rate [138,143], and this hyperactivity has also been found in the offspring of fish exposed to PFOS [142]. While these multi-generational effects are believed to be caused by direct oviparous maternal transfer of PFOS rather than residual chemical exposure, given chemical analysis of maternal vs. paternal body of burden concentrations, the discrepancies in results across published literature highlight the need for future research in this domain to confirm a causal mechanism of transfer and effect [138].

The paucity of studies focused on individual- and population-level effects of PFAS exposure is also reflected by the lack of studies that directly link PFAS exposure with standardized fish health indices such as the hepatosomatic index, gonadosomatic index, and Fulton’s condition factor. In a single study that investigated the effect of environmental levels of PFAS on morphometric fish health indices, it was determined that Fulton’s condition factor was directly affected by PFAS exposure, and the HSI was also directly affected for certain fish species [144]. However, as the study was based on the field collection of fish species and causal substance exposure was determined by environmental sampling, the study was unable to identify the main contributions by individual PFAS [144]. Therefore, we recommend standardized laboratory studies on health indices in fish as another direction of future research for PFAS in general.

### 5.4 Gaps in knowledge and future concerns

The amount of PFAS used in industrial and commercial processes and the growing number of substances detected in the environment are an inherent difficulty associated with any research on this chemical class [1,7]. Discrepancies in exposure periods, model organisms, concentrations of exposure, and chemical of study have made it difficult to rank PFAS in terms of toxicity [11]. While PFOS is generally considered the most toxic PFAS, this assumption is only supported by a small amount of toxicity information on other substances in the environment [11,[24], [25], [26], [27]]. Depending on the endpoint of study, the ranking of substances can change as well. For example, exposure to PFOS but not PFOA at environmentally relevant concentrations resulted in chronic toxicity in *Daphnia carinata* [145], while dose-dependent increases in lipid-peroxidation were observed in tilapia (*Oreochromis niloticus*) only with exposure to PFOA, not PFOS [104]. Additional studies on population- and individual-level effects of PFAS exposure would aid in highlighting the overall effects and general toxicity of substances, while also highlighting potential biological mechanisms of toxicity to be confirmed with future studies.

Large concern also surrounds the mixture toxicity of PFAS chemicals and other micro-pollutants [11]. While PFAS often behave differently in the environment compared to other micro-pollutants [146], evidence suggests exposure to PFAS could impact the toxic potency of other micropollutants in the environment. In a study investigating the combined effect of binary and tertiary mixtures of PFOS with pesticides and/or pharmaceuticals, both antagonistic and synergistic toxic responses were observed [147]. Further, it has been theorized that the immunosuppressive effects of PFAS exposure could make organisms more susceptible to infection and less resilient to environmental stress [11,146]. This has been supported by a study in which exposure to 10 μg/L (10 ppb) of PFHxS increased trematode infections in larval northern leopard frogs compared to the negative control [148]. However, exposure to PFOS did not result in a similar increase in susceptibility, highlighting the gaps in knowledge that exist surrounding PFAS chemicals.

In summary, molecular-level mechanisms such as oxidative stress, nuclear receptor activation, and membrane interaction of PFAS can result in tissue- and organ-level effects that can result in reproductive toxicity, growth and developmental defects, neurobehavioral defects, and other disorders. However, more research is not only needed to highlight the general individual- and population-level effects of exposure but it also elucidate the underlying mechanisms and molecular responses to PFAS leading to such individual- and population-level alterations. ‘Crosstalk’ between the different systems and diverse molecular pathways could be linked with PFAS-induced toxicity and help explain some of the contrasting results observed at both the molecular and individual levels [11,149]. For instance, it has been theorized that oxidative stress can affect the formation of eggs and the development of larvae, relating it to reproductive toxicity [11,129], and PFAS affect the production and regulation of lipids, which can be precursors for sex hormones [11,118,123]. While such systematic interactions could help clarify the adverse effects related to PFAS exposure, the field of PFAS-induced toxicity also suffers from unidentified fluorinated chemicals, lack of toxicity information, a deficit of studies using non-teleost models, and a disconnect between available results and environmentally relevant chemical concentrations and scenarios [25,29].

Therefore, we suggest future studies of PFAS should focus on population- and individual-level effects in order to better support a general understanding of PFAS toxicity in the aquatic environment, and specific focus should be placed on determining exposure effects on standardized health indices to allow for better comparison across species. As well, more mixture studies are required to elucidate the effect of PFAS in an environmentally relevant scenario, as well as highlight mechanisms of their toxicity. Finally, investigations using new techniques such as high-throughput omics could also offer further insights into the environmental effects of PFAS exposure.

## 6 Aquatic toxicology of novel, emerging PFAS of concern

While extensive research on the environmental effects of PFOS and PFOA has occurred, critical scientific and policy needs remain. The large number of PFAS on the global market ensures that most of them remain un- or under-assessed and un- or under-regulated, with extensive data gaps in the public domain [25]. This has led to concerns that PFAS research might never converge due to (1) a lack of information on mixture effects, total chemical burden, and mechanisms of action of both the numerous known and unknown chemicals, (2) current technology that might not be sufficient for detecting decreasing concentrations in the environment, and (3) the constant production of alternative substances that are being created and released into the environment [10,150]. However, recent progress has been made in each, particularly in the areas of grouping PFAS chemicals and prioritizing future research needs [22].

**Fig. 2:**
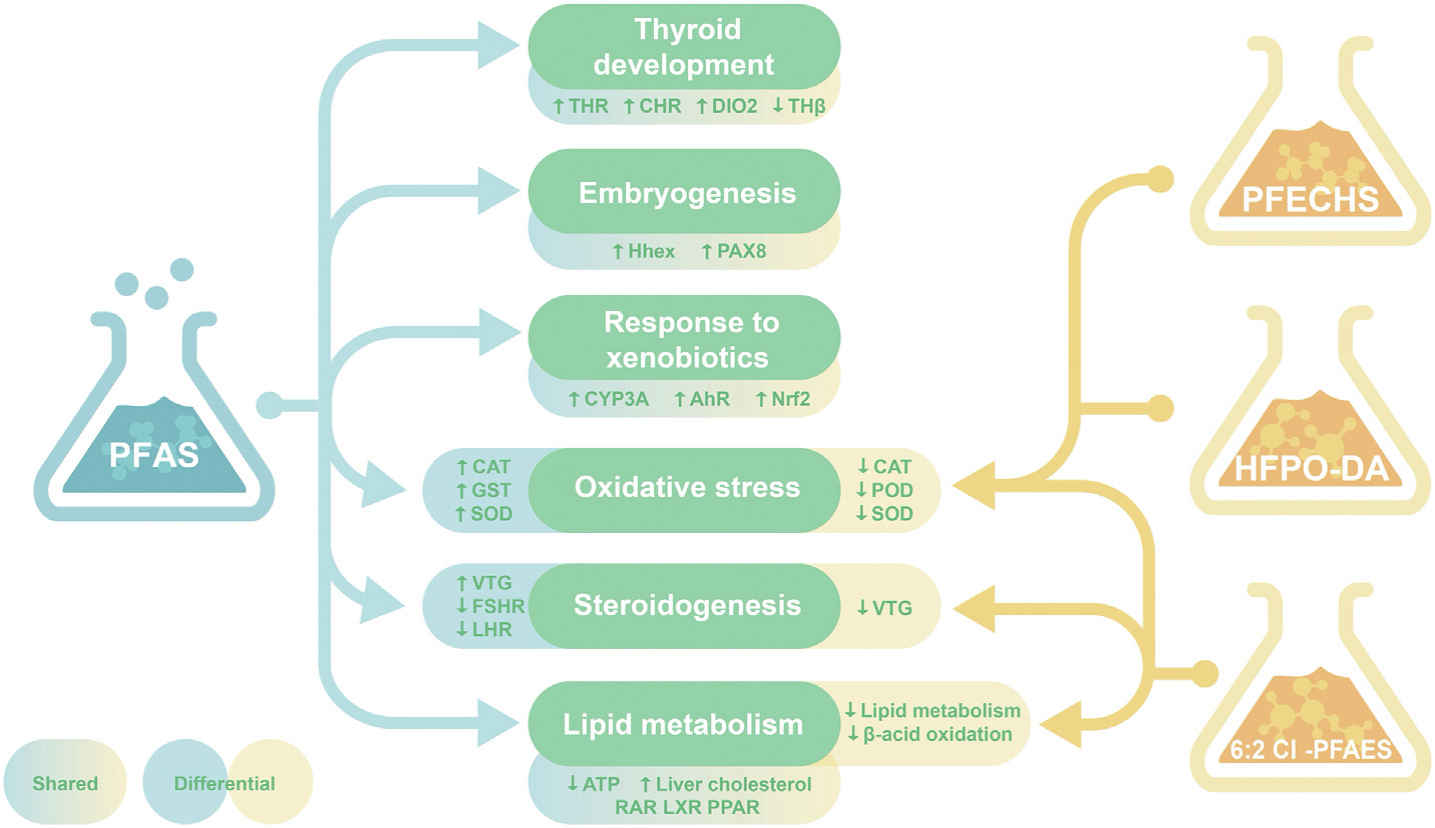
Summary of the most common shared and differential molecular effects between legacy perfluoroalkyl substances (blue) and novel replacement perfluoroalkyl substances (orange). The arrows point to the effects associated with the highlighted compounds.

As knowledge of properties and the ability to define and group PFAS increases, it has become more likely that due to pressure from the scientific and stakeholder communities, governmental and industrial organizations will continue to employ blanket bans on legacy PFAS such as PFOS and PFOA [23,28]. Blanket bans, however, will neither remove the PFAS that already exist in the environment nor will they stop new and related PFAS chemicals from being produced and emerging as aquatic contaminants. Therefore, the following sections will outline the known toxicological information of the chosen replacement PFAS: HFPO-DA, 6:2 Cl-PFAES, and PFECHS and summarize the information in comparison to that known of legacy PFAS (Fig. 2).

### 6.1 Hexafluoropropylene Oxide Dimer (HFPO-DA)

Most toxicological research on the GenX class exists for HFPO-DA, the final product detected in aquatic environments [44,45]. As a shorter chained PFAS (≤6 carbons), HFPO-DA has been marketed as a safer alternative to other PFAS used historically and has been incorporated by many industries in recent years [40,44]. However, the detection of HFPO-DA in surface waters and other environments indicated concern for its safety, and subsequent toxicological studies indicated that HFPO-DA was potentially as toxic, if not more, as the previous legacy PFAS it was meant to replace [139,165,166]. Significant concern arose surrounding human health implications after HFPO-DA was shown to be carcinogenic and toxic in rats and mammals [165,167]. However, relatively little is known about its impact in the aquatic environment and on aquatic organisms [11].

Most studies of HFPO-DA have focused on reproductive, development, growth, and mortality endpoints after aqueous and dietary exposure to HFPO-DA in zebrafish, rainbow trout, common carp, algae, and *D. magna* [18]. In a 12-day study involving the exposure of HFPO-DA to the algae *C. pyrenoidosa,* growth was inhibited after 6 days, and RNA-seq analysis showed that genes related to photosynthesis were downregulated in response to HFPO-DA at concentrations of 100 ng/L and 100 μg/L [168]. Differentially expressed genes were related to photosystem I and photosystem II proteins necessary for the photosynthetic pathways [168]. Similar studies have also shown that HFPO-DA inhibited the antioxidant capacity of algae and increased the production of the ROS indicated by a reduction in cellular chlorophyll contents at concentrations higher than 25 mg/L, as well as differential transcription of genes related to the oxidative stress pathway and photosynthesis, such as CAT, SOD, and GST [169]. These molecular-level impacts can translate to cell-level effects in *Chlorella* sp. such as a reduction in cellular growth at environmentally relevant exposure concentrations of 10, 100, and 1000 ng/L [151].

In vertebrate species, the acute lethal concentration of 50% (LC_50_) of HFPO-DA has been quantified to be >96.9 mg/L in adult rainbow trout [170], and similar results have been observed in medaka exposed to HFPO-DA which has a recorded LC_50_ greater than 100 mg/L [170]. Rare gudgeon (*G. rarus*) have been shown to be less sensitive to HFPO-DA with a recorded LC_50_ greater than 150 mg/L [170]. These acute toxicity values are significantly more potent than those recorded for PFOA. In multiple studies investigating the acute lethality of PFOA to early-life-stage fish, the recorded LC_50_ values were 430 and 730 mg/L for early-life stage zebrafish and rainbow trout, respectively [171,172]. However, PFOA is better known for causing sublethal chronic effects associated with exposure [11,103,142]. As there are little to no published studies on long-term exposure of HFPO-DA at sublethal concentrations, it is not possible to make a reliable statement comparing the overall toxic potency of HFPO-DA to legacy PFAS, although it appears to be more toxically potent at acute levels of exposure.

In fish, HFPO-DA homologs of trimer and tetramer acids have also been shown to exhibit a binding affinity to ligand-binding domains of ERs, with the lowest observable effect concentration for binding being 25 μM (∼0.08 μg/L) and 12.5 μM (∼0.04 μg/L), respectively [173]. While HFPO-DA did not show an ability to bind to ERs, it was shown to affect the expression of fatty-acid binding proteins at concentrations higher than 50 μM [174]. All homologs were concluded to have the potential to alter the sex-hormone balance and enhance the vitellogenin levels [91,173,175]. In a singular bioaccumulation test in common carp, the whole-body BCFs over a 28-day test exposure period were determined to be <30 [170]. Compared to the calculated whole-body BCF of PFOA which was measured to be 200 in carp as well, HFPO-DA has a lesser bioaccumulation potential [81].

While the toxic potency of HFPO-DA compared to legacy PFAS depends on the duration of exposure, species, and endpoints tested, the mechanisms of toxicity appear to be similar. Exposure of longer-chain, legacy PFAS to algae is known to result in the downregulation of SOD and CAT activity in antioxidant systems [117,149,160]. While this was also observed in exposure to HFPO-DA, further effects included the overall downregulation of the algae’s total antioxidant capacity [168,169]. Further, certain homologues of HFPO-DA have a higher binding affinity to ERs than PFOA where the lowest observable effect concentration is 50 μM (∼1.6 μg/L) [173]. While HFPO-DA specifically was not observed to bind to ERs, it was shown to impact the expression of fatty-acid binding protein [174]. Fatty-acid binding proteins are required for the transport of hydrophobic ligands into cells before fatty acid oxidation is able to take place [176]. As described previously, legacy PFAS impact fatty acid oxidation [25,103,117] which can ultimately lead to observed hepatoxic effects [25,82,103,117,149,160].

### 6.2 6:2 Chlorinated Polyfluoroalkyl Ether Sulfonate (6:2 Cl-PFAES)

Initially, 6:2 Cl-PFAES was marketed by manufacturers as less persistent, less bio-accumulative, and less toxic compared to other, greater molecular mass PFAS like PFOS [51]. However, recent evidence suggests that these proclamations are not necessarily true, and 6:2 Cl-PFAES likely poses a significant risk to the health of the aquatic environment [74,77]. Evidence surrounding bioaccumulation of 6:2 Cl-PFAES as well as long-range transport has increased in recent years [71,152]. 6:2 Cl-PFAES has been shown to be bioaccumulative in several species, including algae and fish. It was reported that whole-body log BAF in Crucian carp (*Carassius carassius*) exceeded the regulatory bioaccumulation criterion with log BAF values between 4.1 and 4.3 [74,153], ranking the bioaccumulation potential of 6:2 Cl-PFAES above that of PFOS [56]. 6:2 Cl-PFAES has been detected in the livers of ringed seals, polar bears, and killer whales, mirroring the detection of PFOS in marine and arctic mammals [49,154]. Although detected at concentrations approximately four-fold less than PFOS, the detection of 6:2 Cl-PFAES in keystone species as well as the observed bioaccumulation and maternal transfer in model fish species greatly increases its potential risk for the health of humans and wildlife [152,153,155,156].

In the freshwater algal species *Scenedesmus obliquus* (*S. obliquus*)*,* exposure to 6:2 Cl-PFAES resulted in many toxic effects associated with exposure to PFOS [157]. Exposure to environmentally relevant concentrations caused an oxidative stress response, increased cell membrane permeability, and mitochondrial membrane potential, as well as direct growth toxicity at concentrations similar to or even less than the no-effect level of PFOS [157]. Specifically, exposure to 50 mg/L of 6:2 Cl-PFAES doubled the permeability of the cellular membrane of algae, while previously reported exposure to 30 mg/L of PFOS had the same effect [134]. 6:2 Cl-PFAES was also observed to be more potent at reducing growth in *S. obliquus* than PFOS, with a reported 50% inhibition concentration (IC50) of 40.3 mg/L 6:2 Cl-PFAES compared to an IC50 of 112 mg/L PFOS [134,157]. These results have also been observed in other algae species such as *Chlorella* sp., which demonstrated reduced growth at environmentally relevant concentrations of 6:2 Cl-PFAES, increased SOD and GSH activity, and decreased activities of CAT and peroxidase (POD) [151,152]. In zebrafish, exposure to 6:2 Cl-PFAES has also been shown to have multi-generational effects. Exposure of the parent generation to 6:2 Cl-PFAES has been shown to impair the embryonic development of offspring by induction of oxidative stress [155], disrupt the expression of HPG-axis genes in both generation one and two offspring, and affect concentrations of thyroid hormone in generation one offspring [156].

Furthermore, in zebrafish, chronic exposure to 6:2 Cl-PFAES at environmentally relevant concentrations resulted in the compound accumulating in the liver, gonads, and embryos [156,157], similar to the accumulation of other PFAS [118,132]. Greater mean concentrations of 6:2 Cl-PFAES were found in the livers of male fish (111.4–67.5 ng/mg), while greater concentrations were found in the gonads of females [158]. This sex-dependent accumulation has also been observed after exposure to other PFAS samples [121,[159], [160], [161]]. Consequently, 6:2 Cl-PFAES has been associated with a greater incidence of liver injury, including hepatomegaly and changes in the pathological structure of the tissue [156,162]. This relates to effects on the liver due to exposure to other long-chain PFAS have on fish, including hepatocellular hypertrophy, cytoplasmic vacuolation, necrosis, and apoptosis [118,163]. 6:2 Cl-PFAES has also been shown to interfere with the PPAR signal pathway in adult zebrafish [155], indicated by down-regulation of genes related to fatty acid β-oxidation (*Acox1,* *Cpt2,* *Cpt1a*), lipid transport (*LPL, CD36*), and cholesterol metabolism (*CYP27A, Nrlh3*) [155], similar to responses observed after exposure to PFOS and PFOA [111,113,164]. Oxidative stress biomarkers such as SOD, CAT, and GSH were also affected by exposure [151]. The observed decrease in SOD and CAT and increase in GSH have been observed in response to long-chain compounds PFOS and PFOA [113,116,118,119].

### 6.3 Perfluoroethylcyclohexane Sulphonate (PFECHS)

Little data is available to characterize the toxic potencies of PFECHS to humans or wildlife, as only two studies exist that characterize its biological effects and toxicities to aquatic organisms [60,151]. The first study investigated the acute and chronic toxic potency of PFECHS to *D. magna,* and the second investigated the effect of PFECHS on the growth and proliferation of *Chlorella* sp. [60,151]. The studies resulted in significantly less growth and inhibited CAT activity, increased SOD and peroxidase activities, and down-regulation of vitellogenin-related genes [60,151]. These results suggest that exposure to PFECHS could result in oxidative stress and endocrine disruption.

In other studies investigating the compartmentalization of PFECHS in field samples, PFECHS has been observed to bioaccumulate in kidney, liver, blood, muscle, and plasma of fish [56,60]. The log BAF of PFECHS has been estimated to be 2.7 [56] and 2.8 [55], ranking below PFOS, which has log BAFs ranging from 3.6 to 4.6 depending on whether it is branched or linear [56]. However, the liver/blood partitioning ratio of PFECHS in fish is estimated to be significantly greater than that of PFOS, and PFECHS and PFOS likely share similar mechanisms of uptake and distribution [56].

The LC_50_ of PFECHS was estimated to be 186.61 mg/L when exposed to *D. magna* for 48 h [60]. This high LC_50_ is supported by a following study where it was determined that PFECHS did not have an effect on *Chlorella* sp. growth rates at concentrations below 1000 ng/L, much higher than its environmental concentrations [151]. Both studies suggested that PFECHS has a lower toxic potency than PFOS, which has calculated EC50 values typically less than 150 mg/L for growth endpoints in various invertebrate species [27]. However, as discussed throughout this review, the toxicity of legacy PFAS can differ significantly between species of exposure [27,60,151]. The toxic potency of PFAS can be significantly higher in fish species than invertebrates, particularly at sensitive times of development, as exemplified by Shi et al., in which the approximate 96-h LC_50_ for zebrafish embryos was calculated to be less than 1 mg/L [127]. Therefore, it is difficult to accurately compare PFECHS to legacy PFAS until more toxicity information is available. While the limited information on molecular-level effects suggests PFECHS could impact endocrine functions and induce oxidative stress similar to legacy PFAS, whether or not exposure will result in similar cell-, organ-, and individual-level impacts remains unanswered [72].

### 6.4 Gaps in knowledge compared to legacy PFAS

Knowledge of these three novel, emerging PFAS in the environment is limited in the same ways that knowledge of legacy PFAS is limited. There exists little to no studies on individual- and population-level effects, while some investigating molecular-level alterations are available [56,60,151,155,168,169], cell- and tissue-level effects are also limited [156,162,174]. Without a more robust understanding of the toxic effects of exposure, it is not only difficult to understand the true impact of these chemicals in the environment but also the true mechanisms of action associated with their exposure. However, apart from the limitations that apply to PFAS in general, the emerging chemicals also face specific limitations.

While the results surrounding the toxicity of HFPO-DA appear to be related to the toxic mechanisms of other PFAS, studies in fish are limited to a few species, partial-life stage tests, or early-life stages [18]. No studies were published at the time of this review that investigated long-term, chronic effects of HFPO-DA and its related compounds at sublethal concentrations in aquatic organisms. As well, there is relatively little information on the extent of HFPO-DA in the environment. While it is considered highly likely that HFPO-DA is able to follow similar long-range transport as legacy PFAS [31,32,46,48,49], this has yet to be confirmed by environmental sampling from remote environments.

Further, while more papers exist outlining the toxicity of 6:2 Cl-PFAES in the aquatic environment than PFECHS, further investigations are required to clarify the bioaccumulation, environmental fate, and ecotoxicity of this compound in laboratory settings [77]. Environmental variation between matrices and concentrations along with local contamination increasing exposure estimates could introduce biases, affecting results [77].

Finally, PFECHS is inherently limited by the number of studies on its toxicity with two studies investigated its molecular impacts on field-obtained fish and growth endpoints in invertebrates [56,151]. Considering that some physicochemical properties are shared between PFECHS and PFOS, studies investigating the effects of PFECHS on more aquatic organisms are required to obtain a more robust picture of its impact in the environment. Particularly, studies investigating cell- and individual-based effects could give a better overall picture of apical effects of exposure. Given the detection of PFECHS in multiple environmental media around the globe, such information could also help overcome some of the limitations inherent in PFECHS detection. For example, methods for the identification and quantification of PFAS within drinking water sponsored by the USEPA do not include PFECHS as an analyte [177,178].

A major limitation that applies to all novel replacement chemicals is the lack of native standards [57,77,78]. Many replacements are not well characterized physicochemically or isometrically, and impurities associated with the production process of these PFAS can make isolating them difficult [57]. Not only does this limit the ability to track these substances and their isomers in the environment but it also limits the ability to determine exposure concentrations, compartmentalization, and accumulation of the PFAS [57]. Overall, future directives of studies on novel replacement PFAS in the environment should focus on generally identifying cell-to population-level effects, while also following lines of inquiry important for legacy PFAS in general such as mixture effects [11]. However, it is particularly important for future studies to investigate the environmental fate and transport processes of the novel PFAS, particularly for chemicals like HFPO-DA in which *in situ* results support the potential for long-range transport but there is no field evidence identifying its presence in remote locations [31,32,46,49]. Clarifying transport potential, as well as whether global environmental concentrations are conflated by local contamination, is an important research directive for these emerging replacement PFAS.

## 7 Characterizations of risk

Currently, regulations pertaining to the registration of new chemicals in the EU under REACH, the USEPA, and with the Government of Canada require substances to be reported based on the total amount of chemicals produced or utilized per year [179], and the manufacturer or industry in which they are being sent to or used by Government Notices [20]. However, chemicals released or used in small amounts, such as less than 1 t, annually, as is the case for multiple PFAS compounds, are exempt from registration, even if they are associated with adverse environmental and health effects [179]. Therefore, the existence of a toxic chemical registry is not always a prerequisite to indicating the toxic potential of an emerging substance. For this reason, to score the toxicity of the replacement PFAS discussed in this review, the Chemical Scoring and Ranking Assessment Model (SCRAM) was utilized [180]. While multiple other chemical scoring and ranking systems are available for use, such as quantitative structure-activity relationships models [181], we chose to use SCRAM as it had previously been utilized to rank chemicals similar to PFAS [180] and offered a robust uncertainty ranking system which is important for chemicals that lack available toxicity information, as is the case with many PFAS [29].

SCRAM was developed as a tool to standardize the ranking of chemicals of concern among countries and regulatory bodies in which consensus of relevant definitions, guidelines, and toxicity profiles is often disparate [180]. The model is designed to give relative scores and also score uncertainty due to missing or uncertain information on a particular substance. SCRAM includes values for parameters (Table S1), including bioaccumulation, persistence, and toxicity across receptors, eventually outputting a final composite score, in which a higher score is associated with a more potentially environmentally relevant compound [180]. For each parameter, the maximum score achievable is 5, and the uncertainty score can be as high as 5 depending on whether no data are available or predicted data are used [180]. The final chemical, uncertainty, and composite scores are calculated as weighted percents of their associated bioaccumulation, persistence, and toxicity components as described in Part IV of Snyder et al. [180]. Therefore, the lowest potential composite score is 1, which means at least one parameter must be completed for the model to function [180]. For the purposes of this review, HFPO-DA, 6:2 Cl-PFAES, and PFECHS were ranked according to SCRAM and related to PFOS and PFOA to quantify their relative significance in the human and environmental sectors.

The scoring of the SCRAM model ranks each chemical with an overall, composite score that can be used to rank chemicals according to their effect or potential effect in the environment [180]. The composite score increases if the chemical and uncertainty score increase, but chemicals with high uncertainty scores may lead to high composite scores even if the associated chemical score is low. Therefore, this review ranks the chemicals by both their chemical and uncertainty score to avoid potential conflation between which chemicals are most potentially toxic (indicated by a high chemical score) and which chemicals are the best candidates for future research (indicated by a high uncertainty score).

According to the chemical score of each PFAS tested, the ranking from greatest to least potentially toxic was as follows: PFOS > 6:2 Cl-PFAES > PFOA > HFPO-DA > PFECHS (Table S1). While it was not surprising that PFOS remained the most potentially toxic PFAS given the amount of literature on its effects of exposure, what was concerning was the ranking of 6:2 Cl-PFAES above PFOA, indicating its potential to be more acutely toxic. However, this ranking could be affected if more sub-lethal chronic 6:2 Cl-PFAES exposure studies are released, as there is still a small amount of information on chronic aquatic toxicity of 6:2 Cl-PFAES. As well, while HFPO-DA was ranked below that of PFOA for potential toxicity, the SCRAM model only took into consideration its chronic toxicity scores based on its environmental persistence (Table S2). Based on acute toxicity, HFPO-DA is considered to be potentially more toxic than PFOA in certain exposure scenarios [171,172].

When ranked by uncertainty scores, the order for which chemical is a candidate for future research on its toxicity from the highest necessity to the lowest is as follows: PFECHS > HFPO-DA > 6:2 Cl-PFAES > PFOS > PFOA (Table S1). This ranking simply illustrates which chemicals have the least associated amount of toxicity and environmental fate data, of which PFECHS has the lowest. HFPO-DA and 6:2 Cl-PFAES have a similar uncertainty score (13 vs. 12), illustrating all three emergent compounds in this review remain largely uncertain relative to PFOS and PFOA as expected. Based on the results of SCRAM, future studies should focus on evaluating the impact of PFECHS, HFPO-DA, and 6:2 Cl-PFAES in the environment to accurately compare them to legacy chemicals like PFOA and PFOS, and better inform whether replacement PFAS are a viable pathway for future PFAS management strategies.

## 8 Conclusions

Several PFAS chemicals have been removed from the general market in multiple countries or by various industries, and regulations will likely continue to expand to cover more substances and become more encompassing [22,28]. Apart from the significant threat these substances continue to pose to aquatic environments due to their persistence, concern also surrounds the development of replacement compounds, which have also started to appear in various environmental matrices [77]. Preliminary results of a relatively small number of aquatic toxicity studies have suggested that some of the most popular replacements: PFECHS, 6:2 Cl-PFAES, and HFPO-DA, as highlighted in this assessment, potentially pose significant risks to the environment, similar to the legacy substances that they have been developed to replace. The available literature indicates these replacement compounds affect aquatic organisms by causing oxidative stress and dysregulation of genes related to fatty acid β-oxidation and cholesterol metabolism, similar as seen to the effect mechanism of PFOS and PFOA [11].

However, the paucity of toxicity studies on replacement compounds means that there is no robust set of data upon which to base assessments, including information on targeted molecular effects after exposure and a limited number of multi-generational and full-life cycle studies. As well, the lack of reliable detection methods and uncertainty in their environmental spread could impact the understanding of how diverse these chemicals are. The SCRAM model was effective at quantitatively ranking the hazards posed by the three chemicals as well as describing and quantifying uncertainties associated with the ranking so that data gaps could be identified for each compound. Overall, these knowledge gaps in replacement PFAS largely parallel the gaps relating to the aquatic toxicity of PFAS in general. However, given the probability these compounds will emerge in the environment as the contaminants of the future as they replace legacy substances in industrial production, increased focus and scrutiny should be placed on emerging PFAS alternatives, and robust toxicity profiles completed by multiple independent agencies should be determined before global scale marketing.

## Declaration of competing interests

The authors have declared no conflicts of interests.
