# Supplementary material for: Next generation per- and poly-fluoroalkyl substances: Status and trends, aquatic toxicity, and risk assessment
Source: Eco Environ Health. 2022 Jul 19;1(2):117–31. doi: 10.1016/j.eehl.2022.05.002 (PMC10702929; doi:10.1016/j.eehl.2022.05.002)
Supplement: Multimedia component 1 [file mmc1.docx]

Table S1: Values of parameters input into the Chemical Scoring and Ranking Assessment Model (SCRAM) to rank the three Perfluoroalkyl Substance replacements

| **Compound or**  **Group of isomers** | **Parameter** | **Indicator** | **Value** | **Reference** | **SCORE** | | |
| --- | --- | --- | --- | --- | --- | --- | --- |
|  |  |  |  |  | Chemical | Uncertainty | Composite |
| **HFPO-DA** |  |  |  |  | 18 | 13 | 31 |
|  | Bioaccumulation | BAF | 1–100* | [1,2] |  |  |  |
|  | Environmental Persistence | Half-life in water | >5 days | [3] |  |  |  |
|  |  | Half-life in air | >37 years |  |  |  |  |
|  |  | Half-life in biota | 4–20 days |  |  |  |  |
|  |  | Hal-life in sediment | 5 days* |  |  |  |  |
|  |  | Half-life in soil | 5 days* |  |  |  |  |
|  | Acute Terrestrial Toxicity | Chemical score ≥2, therefore acute toxicity was not included in the scoring | | |  |  |  |
|  | Acute Aquatic Toxicity |  |  |  |  |  |  |
|  | Chronic Terrestrial Toxicity | Plants | N/A |  |  |  |  |
|  |  | Mammals | NOAEL  ≤0.1 mg/(kg∙day) | [3,4] |  |  |  |
|  |  | Reptiles/Amphibians | N/A |  |  |  |  |
|  |  | Birds | N/A |  |  |  |  |
|  |  | Invertebrates | N/A |  |  |  |  |
|  | Chronic Aquatic Toxicity | Plants | N/A |  |  |  |  |
|  |  | Amphibians | N/A |  |  |  |  |
|  |  | Warm Water Fish | N/A |  |  |  |  |
|  |  | Cold Water Fish | N/A |  |  |  |  |
|  |  | Invertebrates | N/A |  |  |  |  |
|  | Chronic Human Toxicity | General Toxicity | NOEL >0.5 mg/(kg∙day) | [3] |  |  |  |
|  |  | Reproductive Toxicity | LOEL 5 mg/(kg∙day) |  |  |  |  |
|  |  | Developmental Toxicity | LOEL  10 mg/(kg∙day) |  |  |  |  |
|  |  | Carcinogenicity | N/A |  |  |  |  |
| **6:2 CL-PFAES** | Bioaccumulation | log Kow | 5.13* | [5]  CompTox ID: DTXSID60881236 | 33 | 12 | 45 |
|  |  | BAF | 25,118 | [6] |  |  |  |
|  |  | BCF | 3612–251,188* | [8, 5]  CompTox ID: DTXSID60881236 |  |  |  |
|  | Environmental Persistence | Half-life in water | 4 to 20 days | [7] |  |  |  |
|  |  | Half-life in air | 4.62 days* | CompTox ID: DTXSID60881236 |  |  |  |
|  |  | Half-life in biota | 2.76–24 days | [8,9] |  |  |  |
|  |  | Hal-life in sediment | 4.62 days* | CompTox ID: DTXSID60881236 |  |  |  |
|  |  | Half-life in soil | 4.62 days* | CompTox ID: DTXSID60881236 |  |  |  |
|  | Acute Terrestrial Toxicity | Chemical score ≥2, therefore acute toxicity was not included in the scoring | | |  |  |  |
|  | Acute Aquatic Toxicity |  |  |  |  |  |  |
|  | Chronic Terrestrial Toxicity | Plant | N/A |  |  |  |  |
|  |  | Mammals | NOEL  <5 mg/(kg∙day) | [10] |  |  |  |
|  |  | Reptiles/Amphibians | N/A |  |  |  |  |
|  |  | Birds | NOEL 0.004 mg/(kg∙day) | CompTox ID: DTXSID60881236 |  |  |  |
|  |  | Invertebrates | N/A |  |  |  |  |
|  | Chronic Aquatic Toxicity | Plants | LOEC  <5.42 mg/L | [11] |  |  |  |
|  |  | Amphibians | N/A |  |  |  |  |
|  |  | Warm Water Fish | LOEC  <0.05 mg/L | CompTox ID: DTXSID60881236 |  |  |  |
|  |  | Cold Water Fish | N/A |  |  |  |  |
|  |  | Invertebrates | N/A |  |  |  |  |
|  | Chronic Human Toxicity | General Toxicity | LOEC  0.04 mg/(kg∙day) | [12] |  |  |  |
|  |  | Reproductive Toxicity | LOEC  0.5 mg/(kg∙day) | [12] |  |  |  |
|  |  | Developmental Toxicity | LOEC 0.5 mg/(kg∙day) | [13] |  |  |  |
|  |  | Carcinogenicity | N/A |  |  |  |  |
|  |  | Other Toxicity | 0.15 mg/L leads to moderate reversible effects | [14] |  |  |  |
| **PFECHS** | Bioaccumulation | Log Kow | 3.19–5.12* | [2,15]  CompTox ID: DTXSID70275965 | 17 | 32 | 49 |
|  | Environmental Persistence | Half-life in water | 4 to 20 days* | [15]  CompTox ID: DTXSID70275965 |  |  |  |
|  |  | Half-life in air | N/A |  |  |  |  |
|  |  | Half-life in biota | N/A |  |  |  |  |
|  |  | Hal-life in sediment | N/A |  |  |  |  |
|  |  | Half-life in soil | N/A |  |  |  |  |
|  | Acute Terrestrial Toxicity | Plants | N/A |  |  |  |  |
|  |  | Mammals | N/A |  |  |  |  |
|  |  | Birds | N/A |  |  |  |  |
|  |  | Reptiles/Amphibians | N/A |  |  |  |  |
|  |  | Invertebrates | N/A |  |  |  |  |
|  | Acute Aquatic Toxicity | Plants | LC_50_  >1 mg/L** | [16] |  |  |  |
|  |  | Amphibians | N/A |  |  |  |  |
|  |  | Warm Water Fish | N/A |  |  |  |  |
|  |  | Cold Water Fish | N/A |  |  |  |  |
|  |  | Invertebrates | LC_50_  186.1 mg/L* | [17] |  |  |  |
|  | Chronic Terrestrial Toxicity | Chemical score ≤2, therefore chronic toxicity was not included in scoring | | |  |  |  |
|  | Chronic Aquatic Toxicity |  |  |  |  |  |  |
|  | Chronic Human Toxicity |  |  |  |  |  |  |
| **PFOS** | Bioaccumulation | BC | 200–1500 | PubChem CID 74483; | 37 | 6 | 43 |
|  |  | BAF | 3500 | [18] |  |  |  |
|  | Environmental Persistence | Half-life in Biota | 8.2–20.4 days in trout;  8.76 years in human males | PubChem CID 74438 |  |  |  |
|  |  | Half-life in air | 115 days |  |  |  |  |
|  |  | Half-life in soil | >41 years* |  |  |  |  |
|  |  | Half-life in sediment | >41 years* |  |  |  |  |
|  |  | Half-life in water | >41 years* |  |  |  |  |
|  | Acute Terrestrial Toxicity | Chemical score ≥2, therefore acute toxicity was not included in the scoring | | |  |  |  |
|  | Acute Aquatic Toxicity |  |  |  |  |  |  |
|  | Chronic Terrestrial Toxicity | Plants | NOEC 50 mg/L | [19] |  |  |  |
|  |  | Mammals | NOEL  1 mg/kg | [20] |  |  |  |
|  |  | Reptiles/Amphibians | N/A |  |  |  |  |
|  |  | Birds | NOAEL 0.58 mg/kg/day | [21] |  |  |  |
|  |  | Invertebrates | NOAEL  <1 mg/L | [22] |  |  |  |
|  | Chronic Aquatic Toxicity | Plants | LOAEC 1.2 ug/L | [23] |  |  |  |
|  |  | Amphibians | LOAEC 3.0 mg/L | [24] |  |  |  |
|  |  | Warm Water Fish | NOAEC  <1.2 ug/L | [23] |  |  |  |
|  |  | Cold Water Fish | NOAEC  <7.8 mg/L | [22] |  |  |  |
|  |  | Invertebrates | NOEC  5 mg/L | [19] |  |  |  |
|  | Chronic Human Toxicity | General Toxicity | NOAEL  0.5 mg/(kg∙day) | [25,26] |  |  |  |
|  |  | Reproductive Toxicity | NOAEL:  3.2 mg/(kg∙day) | [27] |  |  |  |
|  |  | Developmental Toxicity | NOAEL:  0.4 mg/(kg∙day) | [27] |  |  |  |
|  |  | Carcinogenicity | Effective Dose for 10% increase in tumors:  8 mg/(L∙day) | [27] |  |  |  |
| **PFOA** | Bioaccumulation | BCF | 5.1–230 depending on species | PubChem CID 9554 | 30 | 5 | 35 |
|  |  | BAF | <31.62 | [28] |  |  |  |
|  | Environmental Persistence | Half-Life in Biota | 12.6 days in rainbow trout;  2.4 years in humans | PubChem CID 9554 |  |  |  |
|  |  | Half-Life in Air | 31 days |  |  |  |  |
|  |  | Half-Life in Soil | 6 months* | [29] |  |  |  |
|  |  | Half-Life in Sediment | 6 months* |  |  |  |  |
|  |  | Half-Life in Water | >25,000 years* | [30] |  |  |  |
|  | Acute Terrestrial Toxicity | Chemical score ≥2, therefore acute toxicity was not included in the scoring | | |  |  |  |
|  | Acute Aquatic Toxicity |  |  |  |  |  |  |
|  | Chronic Terrestrial Toxicity | Plants | NOEC 62.5 mg/L | [28] |  |  |  |
|  |  | Mammals | NOEC  >0.2 ug/mL | [31] |  |  |  |
|  |  | Reptiles & Amphibians | NOEL  <100 mg/L | [32] |  |  |  |
|  |  | Birds | LOEL  <2.5 ug/g | [33] |  |  |  |
|  |  | Invertebrates | NOEC 1 mg/(kg soil weight) | [34] |  |  |  |
|  | Chronic Aquatic Toxicity | Plants | NOEC  12.5 mg/L | [35] |  |  |  |
|  |  | Amphibians | NOEC  <10 ug/L | [36] |  |  |  |
|  |  | Warm-Water Fish | LOEC >3.4 ng/L | [37] |  |  |  |
|  |  | Cold-Water Fish | LOEC  76 mg/L | [38] |  |  |  |
|  |  | Invertebrates | NOEC  <8 mg/L | [38] |  |  |  |
|  | Chronic Human Toxicity | General Toxicity | NOAEL 1.5 mg/(kg∙day) | [39] |  |  |  |
|  |  | Reproductive Toxicity | LOEC  1 mg/(kg∙day) | [40] |  |  |  |
|  |  | Developmental Toxicity | LOEC  <1 mg/(kg∙day) | [41] |  |  |  |
|  |  | Carcinogenicity | LOEC <20 mg/(kg∙day) | [42,43] |  |  |  |

*Predicted value

**The LC_50_ value was predicted to be significantly greater than 1 mg/L from the small effects observed at this level

References

[1] Y. Pan, H. Zhang, Q. Cui, N. Sheng, L.W.Y. Yeung, Y. Guo, Y. Sun, J. Dai, First report on the occurrence and bioaccumulation of hexafluoropropylene oxide trimer acid: an emerging concern, Environ. Sci. Technol. 51 (17) (2017) 9553–9560, doi:10.1021/acs.est.7b02259.

[2] M.H. Li, Toxicity of perfluorooctane sulfonate and perfluorooctanoic acid to plants and aquatic invertebrates, Environ. Toxicol. 24 (1) (2009) 95–101, doi:10.1002/tox.20396.

[3] US EPA, Human Health Toxicity Values for Hexafluoropropylene Oxide (HFPO) Dimer Acid and its Ammonium Salt (CASRN 13252‐13‐6 and CASRN 62037‐80‐3), 2018.

[4] M. Ulhaq, G. Carlsson, S. Örn, L. Norrgren, Comparison of developmental toxicity of seven perfluoroalkyl acids to zebrafish embryos, Environ. Toxicol. Pharmacol. 36 (2) (2013) 423–426, doi:10.1016/J.ETAP.2013.05.004.

[5] J. Newsted, P. Jones, Surfactants, perfluorinated, Encyclopedia of Toxicology 121–123 (2005), doi:10.1016/B0-12-369400-0/00914-5.

[6]G. Munoz, J. Liu, S. Vo Duy, S. Sauvé, Analysis of F-53B, Gen-X, ADONA, and emerging fluoroalkylether substances in environmental and biomonitoring samples: a review, Trends Environ. Anal. Chem. 23 (2019) e00066, doi:10.1016/j.teac.2019.e00066.

[7] V. Mommaerts, A. Hagenaars, J. Meyer, W. de Coen, L. Swevers, H. Mosallanejad, G. Smagghe, Impact of a perfluorinated organic compound PFOS on the terrestrial pollinator Bombus terrestris (Insecta, Hymenoptera), Ecotoxicology 20 (2) (2011) 447–456, doi:10.1007/S10646-011-0596-2/FIGURES/1.

[8] Y. Liu, T. Ruan, Y. Lin, A. Liu, M. Yu, R. Liu, M. Meng, Y. Wang, J. Liu, G. Jiang, Chlorinated polyfluoroalkyl ether sulfonic acids in marine organisms from bohai sea, China: occurrence, temporal variations, and trophic transfer behavior, Environ. Sci. Technol. 51 (8) (2017) 4407–4414, doi:10.1021/acs.est.6b06593.

[9] J. Wang, Y. Pan, Q. Cui, B. Yao, J. Wang, J. Dai, Penetration of PFASs across the blood cerebrospinal fluid barrier and its determinants in humans, Environ. Sci. Technol. 52 (22) (2018) 13553–13561, doi:10.1021/acs.est.8b04550.

[10] G.T. Ankley, D.W. Kuehl, M.D. Kahl, K.M. Jensen, B.C. Butterworth, J.W. Nichols, Partial life-cycle toxicity and bioconcentration modeling of perfluorooctanesulfonate in the northern leopard frog (Rana pipiens), Environ. Toxicol. Chem. 23 (11) (2004) 2745–2755, doi:10.1897/03-667.

[11] D.Y. Zhang, X.L. Xu, Y. Lu, H.Y. Xu, H.M. Yan, The effects of perfluorooctane sulfonate (PFOS) on physiological status and proliferation capacity of *Scenedesmus obliqnus*, Appl. Mech. Mater. 209–211 (2012) 1131–1135, doi:10.4028/www.scientific.net/amm.209-211.1131.

[12] G.H. Dong, Y.H. Zhang, L. Zheng, W. Liu, Y.H. Jin, Q.C. He, Chronic effects of perfluorooctanesulfonate exposure on immunotoxicity in adult male C57BL/6 mice, Arch. Toxicol. 83 (9) (2009) 805–815, doi:10.1007/S00204-009-0424-0/FIGURES/7.

[13] Z. Zeng, B. Song, R. Xiao, G. Zeng, J. Gong, M. Chen, P. Xu, P. Zhang, M. Shen, H. Yi, Assessing the human health risks of perfluorooctane sulfonate by in vivo and in vitro studies, Environ. Int. 126 (2019) 598–610, doi:10.1016/j.envint.2019.03.002.

[14] M.HondaA.MutaT.AkasakaY.InoueY.ShimasakiK.Kannan...Y.OshimaIdentification of perfluorooctane sulfonate binding protein in the plasma of tiger pufferfish Takifugu rubripesEcotoxicol. Environ. Saf.1042014409413.

[15] M.T. Case, R.G. York, M.S. Christian, Rat and rabbit oral developmental toxicology studies with two perfluorinated compouds, Elsevier 20 (2) (2022) 101–109, doi:10.1177/109158180102000207.

[16] Y. Wu, M. Deng, Y. Jin, X. Mu, X. He, N.T. Luu, C. Yang, W. Tu, Uptake and elimination of emerging polyfluoroalkyl substance F-53B in zebrafish larvae: response of oxidative stress biomarkers, Chemosphere 215 (2019) 182–188, doi:10.1016/j.chemosphere.2018.10.025.

[17] M. Houde, M. Douville, M. Giraudo, K. Jean, M. Lépine, C. Spencer, A.O. de Silva, Endocrine-disruption potential of perfluoroethylcyclohexane sulfonate (PFECHS) in chronically exposed Daphnia magna, Environ. Pollut. 218 (2016) 950–956, doi:10.1016/j.envpol.2016.08.043.

[18] L.P. Burkhard, Evaluation of published bioconcentration factor (BCF) and bioaccumulation factor (BAF) data for per- and polyfluoroalkyl substances across aquatic species, Environ. Toxicol. Chem. 40 (6) (2021) 1530–1543, doi:10.1002/ETC.5010.

[19] D.J. Luebker, M.T. Case, R.G. York, J.A. Moore, K.J. Hansen, J.L. Butenhoff, Two-generation reproduction and cross-foster studies of perfluorooctanesulfonate (PFOS) in rats, Toxicology 215 (1–2) (2005) 126–148, doi:10.1016/J.TOX.2005.07.018.

[20] J.L. Butenhoff, S.C. Chang, G.W. Olsen, P.J. Thomford, Chronic dietary toxicity and carcinogenicity study with potassium perfluorooctanesulfonate in Sprague Dawley rats, Toxicology 293 (1–3) (2012) 1–15, doi:10.1016/J.TOX.2012.01.003.

[21] M. Zhang, P. Wang, Y. Lu, X. Lu, A. Zhang, Z. Liu, Y. Zhang, K. Khan, S. Sarvajayakesavalu, Bioaccumulation and human exposure of perfluoroalkyl acids (PFAAs) in vegetables from the largest vegetable production base of China, Environ. Int. 135 (2020) 105347, doi:10.1016/J.ENVINT.2019.105347.

[22] M. Renzi, C. Guerranti, A. Giovani, G. Perra, S.E. Focardi, Perfluorinated compounds: levels, trophic web enrichments and human dietary intakes in transitional water ecosystems, Mar. Pollut. Bull. 76 (1–2) (2013) 146–157, doi:10.1016/J.MARPOLBUL.2013.09.014.

[23] S.A. Beach, J.L. Newsted, K. Coady, J.P. Giesy, Ecotoxicological evaluation of perfluorooctanesulfonate (PFOS). Rev, Environ. Contam. Toxicol. 186 (2006) 133–174, doi:10.1007/0-387-32883-1_5.

[24] S. Vaalgamaa, A.v. Vähätalo, N. Perkola, S. Huhtala, Photochemical reactivity of perfluorooctanoic acid (PFOA) in conditions representing surface water. Science of the, Total Environment 409 (16) (2011) 3043–3048, doi:10.1016/J.SCITOTENV.2011.04.036.

[25] G. Zhao, J. Wang, X. Wang, S. Chen, Y. Zhao, F. Gu, A. Xu, L. Wu, Mutagenicity of PFOA in mammalian cells: role of mitochondria-dependent reactive oxygen species, Environ. Sci. Technol. 45 (4) (2010) 1638–1644, doi:10.1021/ES1026129.

[26] B.J. Tornabene, M.F. Chislock, M.E. Gannon, M.S. Sepúlveda, J.T. Hoverman, Relative acute toxicity of three per- and polyfluoroalkyl substances on nine species of larval amphibians, Integrated Environ. Assess. Manag. 17 (4) (2021) 684–690, doi:10.1002/IEAM.4391.

[27] M. Nordén, U. Berger, M. Engwall, Developmental toxicity of PFOS and PFOA in great cormorant (Phalacrocorax carbo sinensis), herring gull (Larus argentatus) and chicken (Gallus argentites domesticus), Environ. Sci. Pollut. Res. Int. 23 (11) (2016) 10855–10862, doi:10.1007/S11356-016-6285-1.

[28] C. Villaroman, R. Custance, Perfluorooctanoic acid (PFOA), Encyclopedia of Toxicology (2005) 355–358, doi:10.1016/B0-12-369400-0/01059-0.

[29] R.W. Flynn, G. Hoover, M. Iacchetta, S. Guffey, C. de Perre, B. Huerta, W. Li, J.T. Hoverman, L. Lee, M.S. Sepúlveda, Comparative toxicity of aquatic PFAS exposure in three species of Amphibians, Environ. Toxicol.Chem. (2022), doi:10.1002/ETC.5319.

[30] L. Cui, Q.F. Zhou, C.Y. Liao, J.J. Fu, G. bin Jiang, Studies on the toxicological effects of PFOA and PFOS on rats using histological observation and chemical analysis, Arch. Environ. Contam. Toxicol. 56 (2) (2009) 338–349, doi:10.1007/S00244-008-9194-6/TABLES/1.

[31] B.E. Blake, H.A. Cope, S.M. Hall, R.D. Keys, B.W. Mahler, J. McCord, B. Scott, H.M. Stapleton, M.J. Strynar, S.A. Elmore, S.E. Fenton, Evaluation of maternal, embryo, and placental effects in CD-1 mice following gestational exposure to perfluorooctanoic acid (PFOA) or hexafluoropropylene oxide dimer acid (HFPO-DA or GenX), Environ. Health Perspect. 128 (2) (2020), doi:10.1289/EHP6233.

[32] M.B. Macon, L.T.R. Villanueva, K. Tatum-Gibbs, R.D. Zehr, M.J. Strynar, J.P. Stanko, S.S. White, L. Helfant, S.E. Fenton, Prenatal perfluorooctanoic acid exposure in CD-1 mice: low-dose developmental effects and internal dosimetry, Toxicol. Sci. 122 (1) (2011) 134–145, doi:10.1093/TOXSCI/KFR076.

[33] NTP, Toxicology and Carcinogenesis Studies of Perfluorooctanoic Acid Administered in Feed to Sprague Dawley (Hsd:Sprague Dawley SD) Rats, vol. 598, National Toxicology Program Technical Report Series, 2020, doi:10.22427/NTP-TR-598.

[34] K.D. Oakes, P.K. Sibley, K.R. Solomon, S.A. Mabury, G.J. van der Kraak, Impact of perfluorooctanoic acid on fathead minnow (*Pimephales promelas*) fatty acyl-coa oxidase activity, circulating steroids, and reproduction in outdoor microcosms, Environ. Toxicol. Chem. 23 (8) (2004) 1912, doi:10.1897/03-190.

[35] Nordén, M., Berger, U., & Engwall, M. 2016. Developmental toxicity of PFOS and PFOA in great cormorant (Phalacrocorax carbo sinensis), herring gull (Larus argentatus) and chicken (Gallus gallus domesticus). *Environmental Science and Pollution Research International,* 23(11), 10855–10862. https://doi.org/10.1007/S11356-016-6285-1

[36] Villaroman, C., & Custance, R. 2005. Perfluorooctanoic acid (PFOA). *Encyclopedia of Toxicology*, 355–358. https://doi.org/10.1016/B0-12-369400-0/01059-0

[37] Q. Huang, S. Dong, C. Fang, X. Wu, T. Ye, Y. Lin, Deep sequencing-based transcriptome profiling analysis of Oryzias melastigma exposed to PFOS, Aquat. Toxicol. 120 (121) (2012) 54–58, doi:10.1016/j.aquatox.2012.04.013.

[38] X. Shi, Y. Du, P.K. Lam, R.S. Wu, B. Zhou, Developmental toxicity and alteration of gene expression in zebrafish embryos exposed to PFOS, Toxicol. Appl. Pharmacol. 230 (1) (2008) 23–32, doi:10.1016/j.taap.2008.01.043.

[39] Flynn, R. W., Hoover, G., Iacchetta, M., Guffey, S., de Perre, C., Huerta, B., Li, W., Hoverman, J. T., Lee, L., & Sepúlveda, M. S. 2022. Comparative Toxicity of Aquatic PFAS Exposure in Three Species of Amphibians. *Environmental Toxicology and Chemistry.* https://doi.org/10.1002/ETC.5319

[40] Cui, L., Zhou, Q. F., Liao, C. Y., Fu, J. J., & Jiang, G. bin. 2009. Studies on the toxicological effects of PFOA and PFOS on rats using histological observation and chemical analysis. *Archives of Environmental Contamination and Toxicology,* 56(2), 338–349. https://doi.org/10.1007/S00244-008-9194-6/TABLES/1

[41] Blake, B. E., Cope, H. A., Hall, S. M., Keys, R. D., Mahler, B. W., McCord, J., Scott, B., Stapleton, H. M., Strynar, M. J., Elmore, S. A., & Fenton, S. E. 2020. Evaluation of maternal, embryo, and placental effects in CD-1 mice following gestational exposure to perfluorooctanoic acid (PFOA) or hexafluoropropylene oxide dimer acid (HFPO-DA or GenX). *Environmental Health Perspectives,* 128(2). https://doi.org/10.1289/EHP6233

[42] Macon, M. B., Villanueva, L. T. R., Tatum-Gibbs, K., Zehr, R. D., Strynar, M. J., Stanko, J. P., White, S. S., Helfant, L., & Fenton, S. E. 2011. Prenatal perfluorooctanoic acid exposure in CD-1 mice: Low-dose developmental effects and internal dosimetry. *Toxicological Sciences,* 122(1), 134–145. https://doi.org/10.1093/TOXSCI/KFR076

[43] NTP. 2020. Toxicology and carcinogenesis studies of perfluorooctanoic acid administered in feed to Sprague Dawley (Hsd:Sprague Dawley SD) rats. *National Toxicology Program Technical Report Series*, 598. https://doi.org/10.22427/NTP-TR-598
